# Feasibility of EHR-based prescreening for an adjuvant breast cancer trial in a safety-net health system

**DOI:** 10.1093/oncolo/oyag339

**Published:** 2026-08-31

**Authors:** Hussain Alkhafaji, Lindsay C K Reynolds, Melissa Howell, Jerry D Henderson, Dedra Preece, Kari J Teigen, David E Gerber, Kalyani Narra

**Affiliations:** University of Texas Southwestern Medical School, Dallas, TX 75390, United States; Office of Clinical Research, JPS Health Network, Fort Worth, TX, United States; IT Business Intelligence, JPS Health Network, Fort Worth, TX, United States; IT Business Intelligence, JPS Health Network, Fort Worth, TX, United States; Baylor Scott and White Medical Center, Temple, TX, United States; Office of Clinical Research, JPS Health Network, Fort Worth, TX, United States; Harold C. Simmons Comprehensive Cancer Center, University of Texas Southwestern Medical Center, Dallas, TX, United States; Department of Internal Medicine (Division of Hematology-Oncology), University of Texas Southwestern Medical Center, Dallas, TX, United States; Peter O’Donnell Jr. School of Public Health, University of Texas Southwestern Medical Center, Dallas, TX, United States; JPS Oncology and Infusion Center, Fort Worth, TX, United States; Burnett School of Medicine, Texas Christian University, Fort Worth, TX, United States

**Keywords:** safety-net health system, clinical trials, breast cancer, electronic health records, prescreening, implementation

## Abstract

**Introduction:**

Racial and ethnic inequities persist in oncology clinical trial participation, while safety-net health systems may face resource and data limitations that complicate candidate identification. We evaluated the feasibility of an electronic health record (EHR)-assisted prescreening workflow for EMBER-4, a phase 3 adjuvant breast cancer trial, at a safety-net health system.

**Methods:**

Structured Query Language (SQL) queries applied to Epic Clarity identified potential candidates using diagnosis codes, receptor-status codes, endocrine therapy medication records, recent oncology follow-up, and appointment data. Research staff then manually reviewed pathology reports, treatment history, comorbidities, and other protocol criteria to determine full eligibility.

**Results:**

The source population with a diagnosis of breast cancer included 829 patients: 29% Hispanic, 30% Black, 31% non-Hispanic White, and 9.7% others. Primary language was English in 75% and Spanish in 18%. The SQL query identified 123 potential candidates; manual review confirmed 18 eligible patients comprising 67% Hispanic and 44% Spanish language, yielding a positive predictive value of 15%. Clinicians identified 3 additional eligible patients missed by the query, for 21 known eligible patients overall. Eleven declined participation, one did not attend the scheduled clinic visit, and 9 consented. One patient withdrew before randomization and 8 were enrolled. Six enrolled patients were Hispanic, 1 was non-Hispanic White and 1 was Black.

**Conclusions:**

In a safety-net oncology setting, her-assisted prescreening was feasible but required manual verification and clinician involvement. The workflow identified most known eligible patients, although incomplete structured data and low positive predictive value limited its utility as a standalone screening method.

Implications for practice Structured EHR prescreening can support identification of clinical trial candidates in safety-net settings, but it should be integrated with manual chart review and clinician engagement. In this single-site evaluation, the SQL query had a positive predictive value of 15% and identified 18 of 21 known eligible patients (86%). Incomplete structured data contributed to missed eligible patients and required manual verification. Institutions considering similar workflows should assess local data quality, staffing requirements, screening workload, and language-access needs.

## Introduction

Racial and ethnic inequities persist in U.S. oncology clinical trial participation despite longstanding efforts to improve trial access and enrollment.^1^^,^^2^ Black and Hispanic individuals remain underrepresented relative to their representation among people diagnosed with cancer.^3–5^ These disparities can arise at multiple stages of the recruitment pathway, including trial availability, eligibility assessment, clinician referral, and patient participation.^6^^,^^7^

In June 2024, the U.S. Food and Drug Administration (FDA) issued draft guidance describing the form, content, submission timing, and waiver process for Diversity Action Plans under the Food and Drug Omnibus Reform Act of 2022. The Diversity Action Plan guidance remains in draft form.^8^ In December 2025, FDA issued final guidance recommending broader eligibility criteria and enrollment and trial-design practices intended to increase participation of populations that more closely reflect patients likely to use an approved drug.^9^

Safety-net health systems care for large numbers of patients from racial and ethnic minority groups and patients who are uninsured or underinsured.^10^^,^^11^ These populations have often been underrepresented in oncology research.^1^^,^^5^ Safety-net systems are therefore important settings for evaluating strategies to broaden trial availability and recruitment.^11^ However, limited research infrastructure, funding, and operational resources can constrain trial activation and screening.^10^^,^^12^ Trial complexity and costs may create additional barriers to opening oncology trials in these settings.^13^

Recruitment that depends primarily on clinician recognition and referral may miss some potentially eligible trial candidates.^14^^,^^15^ Digital approaches have been developed to support clinical trial recruitment and eligibility screening, including electronic health reherd (EHR)-based systems, machine-learning algorithms, and platforms that use natural language processing to evaluate structured and unstructured clinical data.^15^^,^^16^ Published experience with these technologies in oncologic settings remains limited.^15^ Here, we evaluated the feasibility and implementation outcomherof an EHR-assisted prescreening workflow used to identify potential candidates for EMBER-4, a phase 3 adjuvant breast cancer trial, at a single safety-net health system.

## Methods

### Study setting and design

We conducted a single-site descriptive implementation evaluation of an electronic health record-assisted prescreening workflow at JPS Health Network (JPS), a tax-supported safety-net health system serving Tarrant County, Texas.^17^ According to the 2020 JPS Community Health Needs Assessment, 63% of the JPS inpatient payer mix consisted of patients who were uninsured or covered by Medicare, Medicaid, or other government programs, including JPS Connection.^17^ Tarrant County’s population is approximately 32% Hispanic and 19% Black.^18^ The JPS service area includes residents who face financial, insurance-related, language, transportation, and other barriers to healthcare access.^19^ The JPS Oncology and Infusion Center (OIC) has been continuously accredited by the American College of Surgeons Commission on Cancer since 2008. According to data from the JPS tumor registry for years 2020 to 2024, demographics of breast cancer patients include over 99% women, 36% Hispanic, 29% non-Hispanic White, 27% Black, and 8% Asian and others. At diagnosis, a quarter of these patients with breast cancer had private insurance at diagnosis while 19% were self-pay, 26% had JPS Connection (County Charity) and the rest had Medicare/Medicaid. Primary language is English in 71% and Spanish in 23%. Six percent spoke other languages as their primary language. In 2022, JPS became 1 of 2 network affiliate sites in a Cancer Prevention and Research Institute of Texas Clinical Trials Network Award led by the University of Texas Southwestern Medical Center.^20^ Through collaboration with the other network affiliate, Baylor Scott & White in Temple, Texas, JPS learned of and subsequently activated EMBER-4.

EMBER-4 (NCT05514054) is a phase 3, randomized, open-label trial comparing adjuvant imlunestrant, an oral selective estrogen receptor degrader, with investigator’s choice of standard adjuvant endocrine therapy in patients with resected, estrogen receptor (ER)-positive, human epidermal growth factor receptor 2 (HER2)-negative early breast cancer after 24 to 60 months of prior adjuvant endocrine therapy.^21^ Other key eligibility criteria included a high risk of recurrence based on protocol-defined clinicopathologic features; Eastern Cooperative Oncology Group performance status of 0 or 1; adequate organ function; no prior endocrine therapy for breast cancer prevention; and no protocol-excluding history of another cancer.^21^ The protocol-defined recurrence-risk criteria were either at least 4 positive ipsilateral axillary lymph nodes or 1 to three positive ipsilateral axillary lymph nodes accompanied by a tumor measuring at least 5 cm, a grade 3 tumor, or a grade 2 tumor measuring 2 to 5 cm. Prior neoadjuvant or adjuvant chemotherapy and prior treatment with a cyclin-dependent kinase 4/6 inhibitor or poly(ADP-ribose) polymerase inhibitor were permitted.^21^ The trial opened in October 2022 with a planned enrollment of 8000 patients.^21^

### EHR-assisted prescreening process

In April 2024, JPS implemented an electronic health record (EHR)-assisted prescreening workflow to identify potential candidates for EMBER-4. The workflow used Epic Clarity, the reporting database associated with the Epic EHR, to generate patient lists from structured clinical and administrative data.

The JPS IT Business Intelligence team developed and executed Structured Query Language (SQL) queries in Microsoft SQL Server Management Studio to extract structured patient data from the Epic Clarity database. The queries applied predefined inclusion and exclusion criteria to generate a preliminary cohort of potential trial candidates for subsequent manual eligibility review (Table 1). Because the workflow relied on structured EHR fields, it was designed as a prescreening tool rather than a definitive eligibility assessment.

**Table 1.** Information technology prescreening criteria, manual verification, and key limitations.

Criterion or operational filter	Electronic query approach	Manual verification	Key limitation or interpretation
**Breast cancer cohort**	Breast cancer code (C50.011 to C50.929 or Z85.3), diagnosis window, and prespecified oncology or survivorship provider	Clinical history reviewed as needed	Coding and provider assignment may be incomplete
**ER-positive, HER2-negative disease**	ER-positive code required (Z17.0); ER-negative (Z17.1) and HER2-positive (Z17.31) codes excluded. Absence of a HER2-positive code served as the prescreening proxy for HER2-negative disease	Pathology report review confirmed receptor status	No dedicated structured HER2-negative field; missing codes caused false-negative cases
**No distant metastatic disease**	Secondary malignant neoplasm codes outside regional lymph nodes were excluded	Clinical history, imaging, and pathology reviewed	Code-based exclusion was not a definitive metastatic assessment
**Endocrine therapy exposure and duration**	At least 1 medication record mapped to an endocrine-therapy medication identifier	Active use, 24-60 months of prior therapy, continuity, gaps, and discontinuation were confirmed manually	Medication-list completeness and historical status limited electronic assessment
**Protocol-defined recurrence risk**	Not assessed electronically	Tumor size, grade, lymph node involvement, and other clinicopathologic features were reviewed manually	Key features were contained primarily in scanned pathology reports
**Prior CDK4/6 or PARP inhibitor exposure**	Not assessed electronically	Treatment history reviewed manually	Prior use was allowed and current use not allowed. Medication record update not felt to be reliable
**Performance status, organ function, comorbidities, and other protocol exclusions**	Not assessed electronically	Clinical notes, laboratory results, treatment history, and investigator judgment	Many criteria required narrative information or clinical interpretation
**Preferred language**	Documented preferred language limited to English or Spanish	Availability of approved study materials confirmed	Operational filter based on available study materials; may exclude patients with other preferred languages
**Recent oncology engagement**	At least 1 documented JPS oncology encounter during the preceding 12 months	Upcoming appointments reviewed after eligibility assessment	Operational filter; not a validated measure of future retention or adherence. It was used as a surrogate marker for compliance with endocrine therapy, this assumption may have limitations
**Vital status**	Patients documented as deceased were excluded	Reviewed as needed	Recent updates may not have been entered in structured field but as a free text.

Abbreviations: CDK4/6, cyclin-dependent kinase 4/6; ER, estrogen receptor; HER2, human epidermal growth factor receptor 2; PARP, poly(ADP-ribose) polymerase.

The SQL query identified patients with breast cancer diagnosed between March 1, 2018, and March 31, 2022, who were followed by at least 1 of the 15 prespecified JPS providers in medical oncology, radiation oncology, or the survivorship clinic. Patients were required to have an International Classification of Diseases, Tenth Revision, Clinical Modification (ICD-10-CM) breast cancer code (any C50 code ranging from C50.011 to C50.929) and an ER-positive status code Z17.0. Patients with a history of breast cancer (Z85.3) were also included. Records containing an ER-negative code (Z17.1) or a HER2-positive code (Z17.31) were excluded. HER2-negative status was not identified directly from a dedicated negative-status field; the absence of a HER2-positive code served as an electronic prescreening proxy and was subsequently confirmed through manual pathology review. To reduce inclusion of patients with metastatic disease, the query excluded records containing codes for secondary malignant neoplasms outside regional lymph nodes (C78 and C79 codes). The query also required at least 1 EHR medication record mapped to an Epic medication identifier for endocrine therapy prescribed in the adjuvant setting. The endocrine therapy medication identifiers were anastrozole 146 758, letrozole 146 930, exemestane 147 752, and tamoxifen 145 430. Patients were required to have at least 1 of these medications prescribed in the adjuvant setting and listed as active for at least 24 months after diagnosis. The presence of a medication record served only as a prescreening signal; actual active use, treatment duration, and treatment continuity were determined through manual chart review. Prior CDK4/6 and PARP inhibitor exposure was not assessed electronically. While prior use of CDK4/6 and PARP inhibitors was allowed, ongoing concurrent therapy was prohibited. Given that routine medication records rarely reflect real-time treatment discontinuations with sufficient precision, manual adjudication was required to confirm eligibility.

Because pathology reports were stored in the EHR as scanned portable document format files rather than discrete structured fields, the SQL query could not assess tumor size, histologic grade, axillary lymph node involvement, or other clinicopathologic features used to determine recurrence risk. These variables, along with confirmation of ER-positive and HER2-negative status, were therefore evaluated through manual review of pathology reports. The SQL workflow did not use free-text search or natural language processing.

In addition to trial-related eligibility criteria, the electronic query excluded patients whose documented preferred language was neither English nor Spanish because institutional review board-approved consent forms and study materials were available only in those languages. The query also excluded patients without a documented JPS oncology encounter during the preceding 12 months. This operational criterion was used as a proxy for recent engagement in oncology care and was not a validated measure of future trial retention or protocol adherence. Patients documented as deceased in the EHR were also excluded.

Using these filters, the IT team conducted preliminary batch queries in April and May 2024. The lead research coordinator reviewed the resulting patient lists, and the query was subsequently refined, including the addition of vital-status criteria. On June 7, 2024, the revised query was run once to generate the final electronic prescreening cohort. Research staff then cross-referenced this cohort with the Epic scheduling system to identify patients with an appointment at the JPS Oncology and Infusion Center within the subsequent 12 months. For patients who remained potentially eligible after chart review but lacked an appointment within the trial eligibility window, research staff collaborated with the treating oncologist to coordinate an appropriate follow-up visit.

A research coordinator manually reviewed the EHR of each electronically identified patient to assess full trial eligibility, and the EMBER-4 site principal investigator confirmed the final eligibility determination. Manual review verified the duration and continuity of adjuvant endocrine therapy, including whether treatment had been received for 24 to 60 months. The coordinator also reviewed pathology reports to confirm invasive breast cancer, ER-positive and HER2-negative status, tumor size, histologic grade, axillary lymph node involvement, stage, and other clinicopathologic risk features. Treatment history, performance status, organ function, prior malignancies, metastatic disease, and comorbidities relevant to protocol eligibility were also assessed manually when they could not be determined reliably from the structured query. Throughout the trial-accrual period, research staff and oncology clinicians continued to identify potential candidates during routine clinical care, including patients who had been missed by the electronic query.

For patients considered eligible after manual review, research staff reviewed upcoming clinic schedules and sent an electronic EHR alert to the treating clinician up to 1 week before the scheduled oncology visit. The alert informed the clinician that the patient met the prescreening criteria for EMBER-4 and prompted consideration of a trial discussion during the routine encounter.

Before the scheduled visit, a research staff member contacted the patient to introduce EMBER-4 briefly and explain that the trial could be discussed in greater detail during the upcoming appointment. Spanish-speaking patients were contacted by a Spanish-speaking staff member or with assistance from an on-site Spanish interpreter. When patients requested additional information, research staff provided institutional review board-approved study information and consent materials in English or Spanish before the appointment to allow time for review.

Research staff maintained internal tracking logs documenting patient-contact attempts, whether EMBER-4 was discussed during the scheduled clinic encounter, and whether patients expressed interest or declined participation. Consent and randomization status were obtained from trial records. The research team reviewed biweekly progress reports to monitor recruitment activity and patient disposition.

Implementation outcomes included the number of patients identified by the SQL query, the number undergoing manual eligibility review, the number confirmed eligible, the number of known eligible patients missed by the query but subsequently identified by clinicians, and the numbers who consented and were randomized. Randomized patients were considered enrolled. Positive predictive value was calculated as the number of SQL-identified patients confirmed eligible divided by the total number identified by SQL and manually reviewed. We also calculated the proportion of all known eligible patients identified by the SQL query. Because the complete source population did not undergo exhaustive manual chart review, this measure was not interpreted as a formal sensitivity estimate. Race, ethnicity, preferred language insurance status, and clinical characteristics were summarized descriptively at the available stages of the recruitment funnel.

## Results

### Prescreening outcomes and recruitment funnel

Before activation of EMBER-4, JPS had opened 5 National Clinical Trials Network trials and relied primarily on clinician identification and referral of potential candidates during routine clinic visits. Across these trials, 45 patients had been screened and 1 patient enrolled in January 2024. These historical figures are provided as institutional context and were not used as a comparator for the EHR-assisted workflow. EMBER-4 was the first industry-sponsored clinical trial opened at JPS. The trial opened to enrollment at JPS on June 18, 2024, and closed to enrollment globally on November 12, 2025. Spanish-language consent forms and study materials were available from trial activation. One registered nurse research coordinator at 0.5 full-time equivalent (FTE) and a research associate at 0.5 FTE were dedicated to this trial.

**Figure 1.**
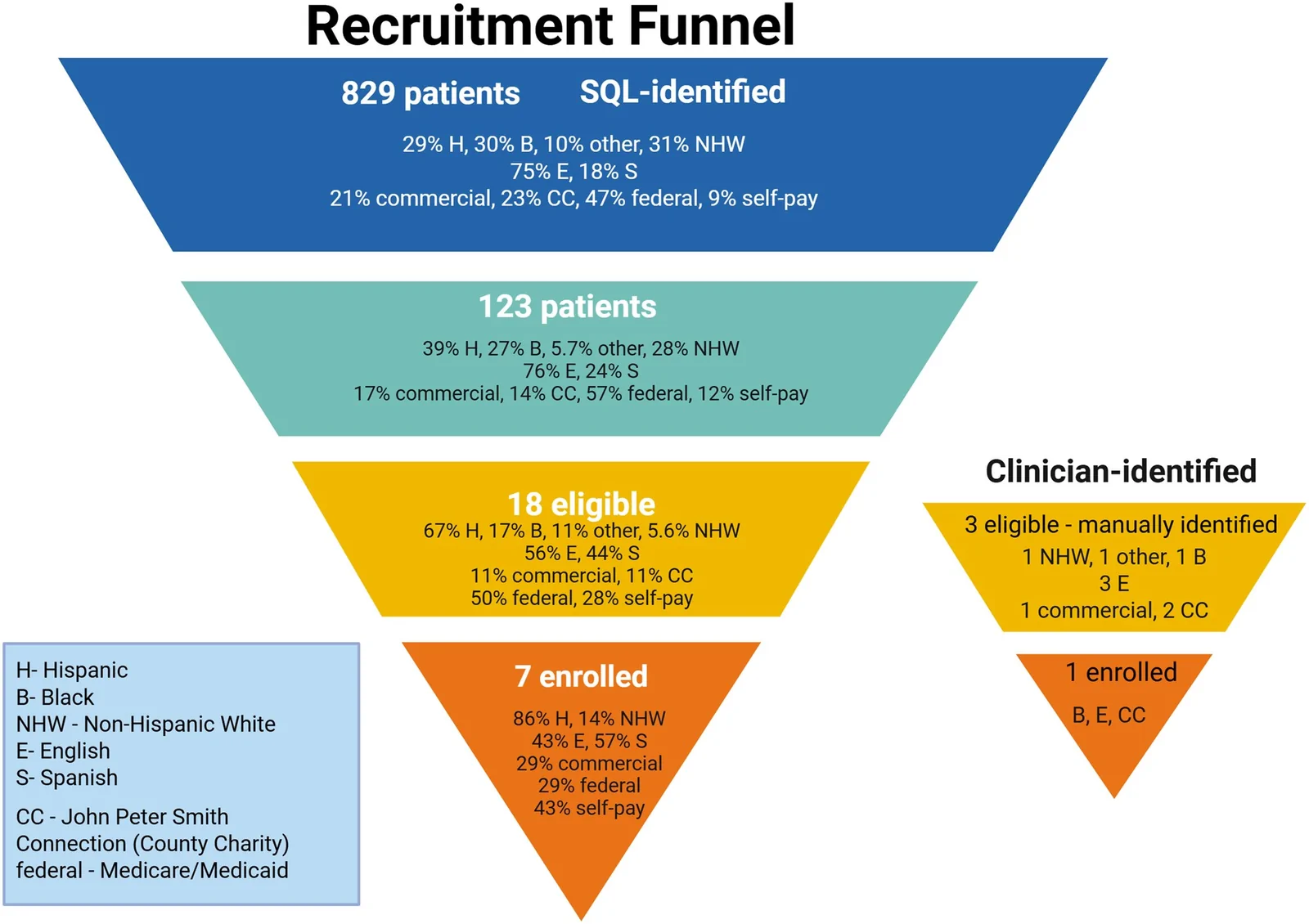
Patient flow through EHR-assisted prescreening, manual eligibility review, consent, and randomization. Patient flow diagram showing 123 patients identified by SQL and manually reviewed; 18 were eligible and 105 were ineligible. Three additional eligible patients were identified through routine clinical care, yielding 21 known eligible patients. Eleven declined, 1 missed the planned encounter, and 9 consented; 1 withdrew before randomization and 8 were randomized.Created with BioRender.com

The total source population that consisted of all breast cancer patients from March 1, 2018 to March 31, 2022 included 829 patients (Figure 1). Racial/ethnic composition was as follows**:** 29% Hispanic, 30% Black, 31% non-Hispanic White and 10% other. Preferred language was English 75%, Spanish 18%, other languages 7%. Insurance mix was as follows: 21% Private insurance, 23% JPS Connection, 47% Medicaid/Medicare and 9% self-pay.

The final SQL query identified 123 potential candidates, all of whom underwent manual eligibility review. These consisted of 39% Hispanic, 27% Black, 28% non-Hispanic White, 6% other. Preferred language was English for 76% and Spanish for 24%. Insurance mix was as follows: 17% Private insurance, 14% JPS Connection, 57% Medicaid/Medicare insurance and 12% self-pay.

Eighteen patients met all trial eligibility criteria, corresponding to a positive predictive value of 15% (18 of 123). Among these 18 SQL-identified eligible patients, 12 were Hispanic, 3 were Black, 8 had Spanish documented as their preferred language, 13 had stage III disease, and 6 had previously received a CDK4/6 inhibitor. Details of these 18 patients are shown in Table 2.

**Table 2.** Characteristics and trial disposition of SQL-identified eligible patients (*N* = 18).

Characteristic	Mean ± SD or *n* (%)
**Age at referral, years**	58 ± 8.7
**Race and ethnicity**	
** Hispanic**	12 (67)
** Black**	3 (17)
** Non-Hispanic White**	1 (5.6)
** Non-Hispanic other**	2 (11)
**Preferred language**	
** English**	10 (56)
** Spanish**	8 (44)
**AJCC anatomic stage^a^**	
** II**	5 (28)
** III**	13 (72)
**Prior CDK4/6 inhibitor**	
** No**	12 (67)
** Yes**	6 (33)
**Enrolled**	
** No**	11 (61)
** Yes**	7 (39)
**Insurance status**	
** Private**	2 (11)
** JPS connection^b^**	2 (11)
** Medicare/medicaid**	9 (50)
** Self-pay**	5 (28)

This table includes only the 18 eligible patients identified by the final SQL query.

Abbreviations: AJCC, American Joint Committee on Cancer; CDK4/6, cyclin-dependent kinase 4/6; SD, standard deviation.

Only includes patients that were on the IT list.

a Stage American Joint Committee on Cancer (AJCC) 8^th^ edition Anatomic Stage.

b Safety-net County Hospital-based medical assistance program for eligible individuals.

Of the 105 SQL-identified patients found ineligible after manual review, 78 (74%) were excluded because of tumor-related criteria. These included insufficient tumor size, grade, or axillary lymph node involvement to meet the protocol-defined recurrence-risk threshold in 71 patients (67%); inflammatory breast cancer histology in 2 (1.9%); and HER2-positive or unknown HER2 status in 5 (4.8%), including 4 with HER2-positive disease and 1 with unknown status. Other reasons for exclusion were an interruption in adjuvant endocrine therapy exceeding 6 months in 2 patients (1.9%), distant metastatic disease in 10 (9.5%), a protocol-excluding cardiovascular condition in 4 (3.8%), and a history of deep venous thrombosis in 11 (11%).

During trial accrual, clinicians identified three additional eligible patients who had not been captured by the final SQL query. Two lacked a documented ER-positive diagnosis code, and 1 had active endocrine therapy that was absent from the EHR medication list. Two of the three missed eligible patients were identified after the final SQL query, and 1 was identified before the SQL query during a clinic visit when our site was in the process of activating the EMBER-4 clinical trial.

Thus, 21 patients were known to meet trial eligibility criteria: 18 identified through SQL and 3 identified outside the electronic query. EHR prompts were sent to clinicians regarding all eligible patients. The SQL query identified 18 eligible patients among 123 potential candidates, giving a positive predictive value of 15%; the remaining 3 were identified through routine clinical care, giving a false negative proportion for the SQL query of 14%.

Among the 21 known eligible patients, 11 declined participation, and 1 additional patient did not attend the previously scheduled clinic visit and 3 phone calls made to reach her to reschedule the missed visit were unanswered. Of the 9 patients who consented, 8 were randomized. The patient who withdrew was SQL identified; she had to go out of the country for an extended period. Of the 8 randomized patients, 7 were identified by SQL and 1 through routine clinical care. Six randomized patients were Hispanic, 1 non-Hispanic White and 1 was Black. All 8 randomized patients were enrolled and started treatment.

Preferred language for all 20 patients that were approached for informed consent was English for 12 and Spanish for 8. Of those 9 patients who signed informed consent, preferred language was English for 4 and Spanish for 5. Of those 8 randomized, English and Spanish were equally represented. Information on interpreter use was not consistently documented to report quantitatively.

Eleven patients were not interested in the study: 1 patient (Hispanic) said that she was skeptical about clinical trials because her cousins had adverse outcomes on clinical trials. When the study was introduced at the clinic visit, 8 patients said they would discuss with family and agreed to be contacted by the research associate 1 week post clinic discussion. During these phone calls, they told the research associate that they were not interested. Two patients deferred decisions for a subsequent clinic visit which happened few months after the initial visit where they deferred decision to enroll, they both eventually declined.

## Discussion

In this single-site implementation evaluation, an EHR-assisted prescreening workflow identified 123 potential candidates for EMBER-4, of whom 18 were confirmed eligible after manual chart review. Clinicians identified 3 additional eligible patients who were missed by the electronic query, resulting in 21 known eligible patients; 9 consented and 8 were randomized. The positive predictive value of the SQL query was 15%, and the query identified 18 of the 21 known eligible patients (86%). In this single-site setting, the EHR-assisted workflow was feasible to implement, but manual verification and continued clinician engagement remained necessary. Eight patients were enrolled: 6 Hispanic, 1 Black, and 1 non-Hispanic White. The proportion of patients of Hispanic ethnicity increased from 29% in the source population to 75% in the enrolled population.

Among the source population, preferred language was English in 75%, Spanish in 18%, and another language in 7%; half of enrolled patients were Spanish-speaking. Because consent forms and study materials were available only in English and Spanish, this operational restriction may have limited trial access for patients with other preferred languages.

Research staff alerted treating clinicians before scheduled visits. Prior studies suggest that clinician communication and trial awareness may influence participation.^22–24^ Standardized electronic screening may reduce dependence on clinician recognition and referral, although this evaluation did not test that effect. Prior work has identified whether clinicians discuss and offer trials as an important interpersonal step in minority trial enrollment.^14^ However, structured electronic screening remains vulnerable to bias because eligibility ascertainment depends on the completeness and accuracy of diagnosis codes, medication lists, and other EHR data. The requirement for an oncology visit within the prior 12 months was an operational filter that may have excluded patients with less consistent engagement in oncology care and introduced selection bias. More broadly, digitally supported recruitment may reproduce existing inequities when access to technology and digital literacy differ across patient populations.^16^^,^^25^

The low positive predictive value primarily reflected eligibility variables that were unavailable in structured EHR fields. Of the 105 SQL-identified patients found ineligible after manual review, 78 (74%) were excluded because of tumor-related criteria, most commonly insufficient tumor size, histologic grade, or axillary lymph node involvement to meet the protocol-defined recurrence-risk criteria. These variables were contained primarily in scanned pathology reports and were not available as discrete structured fields for SQL-based filtering. Manual pathology review was therefore required to determine full eligibility.

The electronic query missed 3 known eligible patients: 2 lacked a documented ER-positive diagnosis code, and 1 had active endocrine therapy that was absent from the EHR medication list. These cases illustrate the dependence of rule-based prescreening on the completeness and accuracy of structured EHR data. Because the complete underlying breast cancer population did not undergo exhaustive manual eligibility review, additional eligible patients may have been missed by both the electronic query and routine clinical care. Therefore, the 18 of 21 known eligible patients identified through SQL represents the proportion of known eligible patients identified by the query and should not be interpreted as formal sensitivity. The true sensitivity of the query cannot be determined from this evaluation.

Electronic identification of potentially eligible patients did not address barriers arising later in the recruitment process. Of the 21 known eligible patients, 11 declined participation and 1 could not be reached. Prior literature has described concerns about treatment side effects, costs, logistical burden, loss of control, and treatment choice, as well as communication-related barriers that may influence trial participation.^6^^,^^14^ The present evaluation did not collect sufficient information to determine whether any of these factors influenced decisions in this cohort. The observed attrition between eligibility confirmation and informed consent illustrates that EHR-assisted prescreening addresses candidate identification but does not, by itself, resolve downstream barriers to participation.

Our workflow represents a pragmatic, rule-based approach within the broader range of digital strategies used for clinical trial recruitment and enrollment. Prior literature has described EHR-based cohort identification, patient-facing digital outreach, social media, research registries, mobile applications, and machine-learning algorithms.^15^^,^^16^^,^^26^ Rule-based EHR recruitment-support systems have been designed to use routinely collected patient data and machine-readable eligibility criteria to generate screening candidates across multiple trials and institutions.^27^ Decentralized trial models, including teletrials, have been proposed as a way to shift some trial activities away from central research sites and potentially expand geographic access.^28^ Prior oncology studies have evaluated automated clinical trial-matching systems using natural language processing and machine learning to assist eligibility screening, including approaches that extract information from unstructured records and compare automated eligibility determinations with manual review.^29^^,^^30^ In contrast, the present workflow used locally developed SQL queries applied to structured Epic Clarity fields for a single trial, followed by manual review of pathology reports and other clinical information. This technically simpler approach may be feasible in settings without specialized natural language processing or artificial intelligence infrastructure, but its performance depends on local coding practices, medication-list completeness, EHR configuration, and staff capacity for manual review. Future studies should evaluate whether searchable pathology data or validated methods for extracting information from unstructured records improve identification accuracy, reduce screening workload, and avoid amplifying biases in the underlying data.

Several limitations should be considered. This was a descriptive implementation evaluation of a single workflow for 1 clinical trial at 1 safety-net health system, involving 21 known eligible patients, 9 patients who provided informed consent, and 8 patients who were randomized. The absence of a prespecified comparison group prevents us from determining whether the workflow improved candidate identification, recruitment efficiency, trial participation, or representation relative to usual recruitment practices. In addition, the complete source population did not undergo exhaustive manual eligibility review; therefore, the number of eligible patients missed by both the electronic query and routine clinical care is unknown, and formal sensitivity could not be calculated. Although all 123 SQL-identified patients underwent manual review, coordinator time, the number of clinician alerts and completed patient contacts, and the number of clinic encounters in which EMBER-4 was discussed were not systematically measured. Consequently, the operational burden and efficiency of the workflow could not be quantified. Operational workflow and staffing requirements may therefore differ across institutions with different EHR configurations, coding practices, data availability, patient populations, and research infrastructure.

## Conclusions

An EHR-assisted prescreening workflow for a complex adjuvant breast cancer trial at a safety-net health system was feasible. The SQL query identified over 80% of the known eligible patients but had a low positive predictive value, missed eligible patients because of incomplete structured data, and required manual chart review and continued investigator engagement. Eight patients were enrolled including patients from racial ethnic minorities. Future evaluations should validate similar workflows against a comprehensive EHR reference standard established by refinement of data extraction guidelines and prospectively measure screening workload, and recruitment outcomes.

## Data Availability

The data underlying this article will be shared on reasonable request to the corresponding author, subject to institutional and regulatory requirements and protection of patient confidentiality.

## Prior presentation

A portion of this work was presented in poster format at the San Antonio Breast Cancer Symposium in San Antonio, Texas, in December 2024.
